# Reference standards for body fat measures using GE dual energy x-ray absorptiometry in Caucasian adults

**DOI:** 10.1371/journal.pone.0175110

**Published:** 2017-04-07

**Authors:** Mary T. Imboden, Whitney A. Welch, Ann M. Swartz, Alexander H. K. Montoye, Holmes W. Finch, Matthew P. Harber, Leonard A. Kaminsky

**Affiliations:** 1 Ball State University, Muncie, Indiana, United States of America; 2 University of Wisconsin-Milwaukee, Milwaukee, Wisconsin, United States of America; TNO, NETHERLANDS

## Abstract

**Background:**

Dual energy x-ray absorptiometry (DXA) is an established technique for the measurement of body composition. Reference values for these variables, particularly those related to fat mass, are necessary for interpretation and accurate classification of those at risk for obesity-related health complications and in need of lifestyle modifications (diet, physical activity, etc.). Currently, there are no reference values available for GE-Healthcare DXA systems and it is known that whole-body and regional fat mass measures differ by DXA manufacturer.

**Objective:**

To develop reference values by age and sex for DXA-derived fat mass measurements with GE-Healthcare systems.

**Methods:**

A de-identified sample of 3,327 participants (2,076 women, 1,251 men) was obtained from Ball State University’s Clinical Exercise Physiology Laboratory and University of Wisconsin-Milwaukee’s Physical Activity & Health Research Laboratory. All scans were completed using a GE Lunar Prodigy or iDXA and data reported included percent body fat (%BF), fat mass index (FMI), and ratios of android-to-gynoid (A/G), trunk/limb, and trunk/leg fat measurements. Percentiles were calculated and a factorial ANOVA was used to determine differences in the mean values for each variable between age and sex.

**Results:**

Normative reference values for fat mass variables from DXA measurements obtained from GE-Healthcare DXA systems are presented as percentiles for both women and men in 10-year age groups. Women had higher (p<0.01) mean %BF and FMI than men, whereas men had higher (p<0.01) mean ratios of A/G, trunk/limb, and trunk/leg fat measurements than women.

**Conclusion:**

These reference values provide clinicians and researchers with a resource for interpretation of DXA-derived fat mass measurements specific to use with GE-Healthcare DXA systems.

## Introduction

Given that obesity (overabundance of fat mass) raises the risk of early mortality and chronic diseases [1–3], high quality body composition measures provided by dual energy x-ray absorptiometry (DXA) have become valued in the clinical and research settings [4]. DXA is a three-compartment method that is considered as a reference technique for measuring body composition (bone, lean, and fat mass), due to its high precision and accuracy compared with other body composition assessments [5,6]. One advantage of DXA is its ability to measure both total and regional body composition through high resolution, high quality imaging [7].

Despite being considered an ideal option for body composition measurement, the interpretation of DXA results is limited by a lack of universally recognized standards for key measures of fat mass variables known to influence health (i.e. % body fat (%BF), android-to-gynoid ratio (A/G ratio), etc.). Recently, Kelly et al. used data from the National Health and Nutrition Examination Survey (NHANES) to develop reference ranges for these measures specific to DXA measurements obtained with the Hologic QDR 4500A fan beam densitometer [4]. Hologic is one of the two major DXA manufacturers, the other being GE Healthcare (Madison, WI; Lunar Prodigy and iDXA models) [5,6,8], which have been validated against criterion 4-compartment models [9,10]. Although both manufacturers use fan beam DXA technology, GE-Healthcare uses narrow-angle, whereas Hologic uses wide-angle fan beam instrumentation. Additionally, body composition results can vary between the devices due to possible differences in calibration standards and specific algorithms used to calculate the body composition measures that are proprietary to the manufacturer [5]. Shepherd et al. compared whole-body body composition results derived using the GE-Healthcare Lunar and Hologic DXA systems, finding significant absolute differences between the two systems in %BF, bone mineral content, and bone mineral density of 1.4%, 176.8g, and 0.013 g/cm^2^, respectively[5]. Others have reported similar differences in body composition variables between these two DXA manufacturers [11–14]. Due to this inter-model variation between DXA manufacturers, the reference values presented by Kelly et al. [4] are only directly compatible with Hologic densitometers and cannot be universally accepted for all DXA models [15].

Researchers have developed cross-calibrated equations between the Hologic and GE-Healthcare models [5]. Fan et al. used these cross-calibrated equations to convert whole-body and regional bone and soft tissue measurements from the NHANES 1999–2004 dataset to reference values for the GE-Healthcare models [16]. Although this study provided an initial set of reference values for body composition measures, including %BF, trunk % fat, legs % fat, and A/G ratio for GE-Healthcare DXAs, researchers reported marginal error associated with the cross-calibrated equations [5]. Therefore, it is important to develop body composition reference values obtained directly from whole-body scans using the GE-Healthcare models. These standards are needed to appropriately determine values for body composition measurements that are associated with an increased risk for chronic diseases.

Other DXA-derived measures of fat mass are useful in evaluating health-risks associated with body composition. Fat mass index (FMI) is a measure of total fat mass divided by height squared, which aids in the interpretation of body composition as it is not confounded by lean tissue as is the case for body mass index (BMI) [4]. Additionally, regional fat mass distribution is an important risk marker for metabolic and cardiovascular health complications [17,18]. When utilizing regional fat mass measurements obtained from DXA, three ratios including the A/G, trunk/limb and trunk/leg have been shown to be good markers of lipodystrophy and correlate with insulin resistance and dyslipidemia [19,20]. Although Kelly et al. developed reference values for FMI, and the A/G, trunk/limb, and trunk/leg ratios, these values are only strictly applicable to the Hologic system [4].

As GE-Healthcare is one of the two major DXA manufacturers [5] widely used by researchers and clinicians, reference values are needed to guide interpretation of body composition results obtained with this instrument. The purpose of this study was to develop reference values for fat mass variables including %BF, FMI, and fat mass ratios of A/G, trunk/limb, and trunk/leg using the GE-Healthcare models.

## Methods

A sample of 3,327 participants, (2,076 women, 1,251 men; 95% of which were Caucasian) was obtained from Ball State University’s Clinical Exercise Physiology Laboratory (2,218 scans) and University of Wisconsin-Milwaukee’s Physical Activity & Health Research Laboratory (1,109 scans). Participants were either self-referred, residents of the surrounding communities, or research subjects or participants in a variety of health-related programs at one of the two laboratories at Ball State University or University of Wisconsin-Milwaukee. All participants were ≥20 and <80 years old (mean age 45.8 ± 18.3 years (**Table 1**)) and for individuals with repeat scans only the results from the first scan were used in the analysis. Participants were excluded if their width exceeded the scanner field or their body weight exceeded the limits of the scanner bed (350 lbs for Prodigy or 450 lbs for iDXA). The study was declared exempt by the Ball State University and University of Wisconsin-Milwaukee IRB as all subject data was de-identified. Additionally, prior to all DEXA scans participants signed an informed consent agreeing that their data from the scan could be used for research purposes.

**Table 1:** Descriptive Characteristics of Participants by Gender (Mean ± SD).

	Men (n = 1,251)	Women (n = 2,076)
**Age (yr)**	46.1 ± 19.1	45.6 ± 17.9
**Ethnicity**	• 92.2% Caucasian	• 96.1% Caucasian
	• 1.3% Black	• 1.2% Black
	• 0.3% Asian	• 0.4% Asian
	• 0.4% Other	• 0.4% Other
	• 5.8% Unspecified	• 1.9% Unspecified
**Height (cm)**	177.8 ± 7.6	164.8 ± 6.9
**Weight (kg)**	89.1 ± 18.6	70.9 ± 18.4
**Bmi (kg/m^2^)**	28.0 ± 5.3	26.4 ± 6.7
**Lean mass (kg)**	61.4 ± 9.7	42.1 ± 6.7

A whole-body DXA scan was performed on all participants. The GE-Healthcare Lunar Prodigy was used at the Clinical Exercise Physiology Laboratory at Ball State University from 2003 to 2010 and the GE-Healthcare iDXA was used from 2010 to October 2015. All scans performed at the Physical Activity & Health Research Laboratory at the University of Wisconsin-Milwaukee used the GE-Healthcare Lunar Prodigy from 2005 to October 2015. Both the GE Lunar Prodigy and GE-Healthcare iDXA use the encore software platform that has been updated as GE-Healthcare releases new versions. These two GE-Healthcare DXA models are both narrow fan-beam densitometers that have high agreement between systems (R^2^ for fat mass = 0.95–0.99), thus are suitable for intra-subject comparisons for %BF [21].

### Procedure

DXA scans were administered by trained research technicians using standardized procedures recommended by GE-Healthcare. All technicians were trained over a period of 1 to 3 months at each laboratory. Prior to each testing session, the GE-Healthcare DXA systems at both laboratories passed the manufacturer recommended quality assurance procedure. Participants were asked to remove all metal, including jewelry and items in their pocket, as well as shoes. Height was measured using a wall-mounted stadiometer and mass was measured with a calibrated scale. The technician then positioned the participant correctly within the scanner field on the DXA table. The final review of all scans was completed by one supervisor at each laboratory.

Variables of interest from the scan that were used in this analysis included %BF, total fat mass, trunk fat mass, leg fat mass, arm fat mass, android fat mass, and gynoid fat mass. The GE-Healthcare systems define the trunk region as including the neck, chest, abdominal and pelvic areas. Its upper perimeter is the inferior edge of the chin and the lower boundary intersects the middle of the femoral necks without touching the brim of the pelvis. The leg region is defined as the point of separation from the pelvic region at an angle perpendicular to the femoral neck. The android region is defined as the area between the ribs and the pelvis that is totally enclosed by the trunk region. The upper boundary is 20% of the distance between the iliac crest and the neck and the lower boundary is at the top of the pelvis. The gynoid region includes the hips and upper thighs and overlaps both the leg and trunk regions[22].

### Statistical analysis

All statistical analyses were performed using SPSS (version 22.0), with descriptive measures reported as means ± standard deviation. Sex- and age-specific body composition measurements were analyzed, with participants classified into age groups by decade (20–29, 30–39, 40–49, 50–59, 60–69, and 70–79 years). Data for both sexes were checked for normality using Kolmogorov-Smirnov test and found to be equally distributed. FMI (fat mass (kg) · height (m⁻^2^)), A/G Ratio (android fat mass/ gynoid fat mass), trunk/leg ratio (total trunk % fat / total leg % fat), and trunk/limb ratio (total trunk fat mass/ [total leg + total arm fat mass]) were calculated from scan measurements. Percentiles were calculated for each outcome variable specific to sex and age groups. A factorial ANOVA was used to determine potential differences in mean %BF values, FMI, A/G ratio, trunk/leg ratio, trunk/limb ratio, between sex- and age-specific groups. An alpha level was set at 0.05 to determine statistical significance. Finally, reference curves were created using LMS regression (S1–S10 Figs)[23]. These curves were fit to the 3^rd^, 50^th^, and 97^th^ percentiles superimposed upon the raw data values. The median values from the NHANES cohort were also added to these curves for comparison.

## Results

Mean (± SD) and percentiles of %BF from the GE-Healthcare models by age for both women and men are displayed in **Table 2**. Women had a greater mean %BF than men in all age groups (women: 38.6 ± 9.9%, men: 28.3 ± 9.4%; p<0.01). The %BF was higher per decade with increasing age up to the 50–59 year age group (p<0.01) in both sexes. Thereafter no differences in %BF were observed in later age groups compared to 50–59 year age group in men. However, in women %BF was lower (P<0.05) in the 70–79 year age group compared to the 60–69 year age group.

**Table 2:** Sex-specific percentiles of body fat percent (%) measured with DXA.

	**Women**										
**Age (yr)**	n	X ± SD	90^th^	80^th^	70^th^	60^th^	50^th^	40^th^	30^th^	20^th^	10^th^
**20–29**	562	31.4± 8.5	22.1	24.5	26.4	28.1	29.9	32.1	35.4	38.3	43.2
**30–39**	196	36.6± 11.0^D^	21.4	26.8	29.3	33.4	36.8	40.6	43.6	46.8	50.7
**40–49**	258	39.2± 9.7^C^	24.5	31.4	34.3	37.4	40.0	42.6	44.5	48.2	51.0
**50–59**	437	41.7± 8.7^B^	30.3	34.4	38.0	40.5	42.7	45.0	47.0	49.3	51.8
**60–69**	440	42.4± 7.7^B^	32.5	35.5	38.6	40.8	42.4	44.9	47.0	49.1	52.2
**70–79**	183	40.4± 7.9^A^	30.5	35.5	37.9	39.6	41.2	43.5	45.8	48.2	50.1
	**Men**										
**Age (yr)**	n	X ± SD	90^th^	80^th^	70^th^	60^th^	50^th^	40^th^	30^th^	20^th^	10^th^
**20–29**	384	21.1±8.3	11.0	14.0	16.5	18.5	20.2	22.7	25.6	28.7	31.8
**30–39**	104	26.3±10.6^D^	11.2	17.2	19.2	23.3	26.4	30.2	33.7	36.6	39.9
**40–49**	145	29.1±8.6^C^	15.8	22.2	25.8	28.4	30.1	31.5	33.9	36.9	41.0
**50–59**	214	30.9±7.9^B^	20.0	24.1	26.8	29.4	31.4	33.7	35.7	37.9	40.8
**60–69**	236	31.0±7.8 ^B^	21.0	25.1	27.3	29.4	31.6	33.5	34.7	37.2	40.7
**70–79**	168	31.1±6.6 ^B^	23.0	25.7	28.0	29.4	32.4	34.0	35.7	37.4	39.6

^A^ Significantly different than 20–29, 30–39, 40–49, 50–59, 60–69; p<0.01.

^B^ Significantly different than 20–29, 30–39, 40–49; p<0.01.

^C^ Significantly different than 20–29, 30–39; p<0.01.

^D^ Significantly different than 20–29; p<0.01.

FMI means (±SD) and percentiles by age group for both women and men are presented in **Table 3**. Mean FMI was higher in women than men at all age groups (women: 10.6± 4.8 kg·m⁻^2^, men: 7.9 ±3.6 kg·m⁻^2^; p< 0.05). Additionally, in both sexes FMI increased with age up until 50–59 year age-group, where it then plateaued before decreasing in the 70–79 year age (p<0.05).

**Table 3:** Sex-specific percentiles of Fat Mass Index (kg·m⁻^2^) measured with DXA.

	**Women**										
**Age(yr)**	n	X ± SD	90^th^	80^th^	70^th^	60^th^	50^th^	40^th^	30^th^	20^th^	10^th^
**20–29**	562	7.6± 3.6	4.3	5.0	5.6	6.0	6.6	7.3	8.2	9.5	11.9
**30–39**	196	10.0±5.2^D^	4.4	5.4	6.4	7.5	8.9	10.2	11.9	14.3	18.0
**40–49**	258	11.0±5.1^C^	4.9	7.0	7.8	8.6	9.7	11.3	12.8	15.4	19.2
**50–59**	437	12.0±4.9 ^B^	6.4	7.6	9.1	10.1	11.3	12.8	14.4	16.3	18.7
**60–69**	440	12.1±4.5 ^B^	6.8	8.0	9.4	10.3	11.2	12.9	14.3	15.6	18.1
**70–79**	183	10.8±3.8^A^	6.4	8.0	8.8	9.6	10.5	11.6	12.8	14.4	16.3
	**Men**										
**Age(yr)**	n	X ± SD	90^th^	80^th^	70^th^	60^th^	50^th^	40^th^	30^th^	20^th^	10^th^
**20–29**	384	5.6±3.0	2.3	3.2	3.8	4.4	5.0	6.0	7.1	8.6	10.7
**30–39**	104	7.3±3.9^D^	2.7	4.0	4.9	5.8	6.8	8.3	10.1	11.0	12.4
**40–49**	145	8.3±3.7^C^	3.6	5.2	6.4	7.3	8.0	8.7	10.5	12.0	14.6
**50–59**	214	8.9±3.4 ^B^	4.7	5.9	6.7	7.8	8.7	9.4	10.3	11.7	13.4
**60–69**	236	8.9±3.4^B^	4.8	6.2	7.0	7.6	8.5	9.2	10.2	11.6	13.6
**70–79**	168	8.4±2.9^A^	5.1	5.8	6.6	7.0	7.9	8.6	9.6	10.7	11.7

^A^ Significantly different than 20–29, 30–39, 40–49, 50–59, 60–69; p<0.05.

^B^ Significantly different than 20–29, 30–39, 40–49; p<0.05.

^C^ Significantly different than 20–29, 30–39; p<0.05.

^D^ Significantly different than 20–29; p<0.05.

Means (±SD) and percentiles of A/G ratio are displayed in **Table 4**. The A/G ratio was different between sexes, with men having a higher mean A/G ratio than women (men: 0.66 ± 0.22, women:0.43 ± 0.17; p<0.01). The mean A/G ratio increased with age in both sexes up until the 60–69 year age-group, (p<0.05) afterwhich there was no difference.

**Table 4:** Sex-specific percentiles of Android-to-Gynoid Ratio measured with DXA.

	**Women**										
**Age (yr)**	n	X ± SD	90^th^	80^th^	70^th^	60^th^	50^th^	40^th^	30^th^	20^th^	10^th^
**20–29**	562	0.33± 0.11	0.20	0.24	0.26	0.28	0.31	0.34	0.36	0.40	0.47
**30–39**	196	0.39±0.17^D^	0.20	0.24	0.29	0.33	0.37	0.40	0.43	0.49	0.62
**40–49**	258	0.44±0.17^C^	0.24	0.29	0.34	0.38	0.41	0.46	0.50	0.56	0.63
**50–59**	437	0.48±0.18^B^	0.28	0.35	0.39	0.43	0.46	0.51	0.55	0.60	0.69
**60–69**	440	0.50±0.15^A^	0.32	0.38	0.42	0.45	0.48	0.52	0.57	0.62	0.72
**70–79**	183	0.46±0.13^A^	0.31	0.36	0.40	0.44	0.47	0.50	0.54	0.59	0.66
	**Men**										
**Age (yr)**	n	X ± SD	90^th^	80^th^	70^th^	60^th^	50^th^	40^th^	30^th^	20^th^	10^th^
**20–29**	384	0.47±0.13	0.29	0.33	0.37	0.40	0.44	0.47	0.49	0.55	0.62
**30–39**	104	0.57±0.15^D^	0.39	0.44	0.47	0.51	0.57	0.61	0.65	0.70	0.79
**40–49**	145	0.66±0.18^C^	0.41	0.50	0.59	0.63	0.68	0.71	0.74	0.80	0.88
**50–59**	214	0.73±0.21^B^	0.48	0.56	0.61	0.66	0.71	0.76	0.81	0.87	0.97
**60–69**	236	0.77±0.20^A^	0.52	0.61	0.67	0.72	0.78	0.82	0.86	0.92	1.01
**70–79**	168	0.76±0.19^A^	0.53	0.61	0.66	0.71	0.77	0.81	0.87	0.98	1.15

^A^ Significantly different than 20–29, 30–39, 40–49, 50–59; p<0.05.

^B^ Significantly different than 20–29, 30–39, 40–49; p<0.01.

^C^ Significantly different than 20–29, 30–39; p<0.01.

^D^ Significantly different than 20–29; p<0.01.

Sex and age group means (±SD) and reference values for trunk/limb fat ratio are displayed in **Table 5**. Men had a higher mean trunk/limb ratio at each age group than women (men: 1.6 ± 0.43, women: 1.1 ± 0.34; p<0.01). Mean trunk/limb ratio was different between the younger age groups (20–29 and 30–39) and the older age groups (60–69 and 70–79) in both sexes (p<0.01).

**Table 5:** Sex-specific percentiles of Trunk-to-Limb Ratio measured with DXA.

	**Women**										
**Age (yr)**	n	X ± SD	90^th^	80^th^	70^th^	60^th^	50^th^	40^th^	30^th^	20^th^	10^th^
**20–29**	562	0.96±0.25	0.71	0.79	0.85	0.88	0.92	0.97	1.03	1.10	1.21
**30–39**	196	1.06±0.34^C^	0.72	0.80	0.86	0.92	1.01	1.06	1.15	1.23	1.48
**40–49**	258	1.12±0.39^C^	0.76	0.86	0.93	0.97	1.03	1.12	1.20	1.31	1.48
**50–59**	437	1.18±0.39^B^	0.79	0.89	0.99	1.05	1.13	1.21	1.30	1.42	1.62
**60–69**	440	1.15±0.29^C^	0.82	0.92	0.98	1.04	1.12	1.19	1.28	1.36	1.55
**70–79**	183	1.12±0.26^C^	0.80	0.89	0.99	1.06	1.12	1.16	1.22	1.30	1.44
	**Men**										
**Age (yr)**	n	X ± SD	90^th^	80^th^	70^th^	60^th^	50^th^	40^th^	30^th^	20^th^	10^th^
**20–29**	384	1.24±0.29	0.85	1.00	1.11	1.18	1.23	1.30	1.36	1.46	1.60
**30–39**	104	1.46±0.28^C^	1.11	1.26	1.33	1.39	1.46	1.54	1.62	1.68	1.80
**40–49**	145	1.65±0.37^B^	1.18	1.35	1.46	1.58	1.63	1.73	1.82	1.96	2.17
**50–59**	214	1.71±0.38	1.24	1.40	1.51	1.59	1.67	1.76	1.85	2.01	2.13
**60–69**	236	1.78±0.44^A^	1.27	1.40	1.51	1.64	1.75	1.85	1.94	2.09	2.25
**70–79**	168	1.75±0.41^A^	1.23	1.32	1.48	1.60	1.70	1.77	1.94	2.07	2.24

^A^ Significantly different than 20–29, 30–39, 40–49; p<0.05.

^B^ Significantly different than 20–29, 30–39, p<0.05.

^C^ Significantly different than 20–29; p<0.05.

Means (± SD) and reference values for trunk/leg fat ratio are displayed in **Table 6**. The mean trunk/leg ratios were greater in men than women across all age groups (men: 1.3 ± 0.42; women: 1.0± 0.23; p<0.01). In both men and women, there were differences in mean trunk/leg ratio in the younger (20–29 and 30–39) compared to older (60–69 and 70–79) age groups. Additionally, there was an increase in trunk/leg ratio between the 60–69 and 70–79 year age groups in women.

**Table 6:** Sex-specific percentiles of Trunk-to-Leg Ratio measured with DXA.

	**Women**										
**Age (yr)**	n	X ± SD	90^th^	80^th^	70^th^	60^th^	50^th^	40^th^	30^th^	20^th^	10^th^
**20–29**	562	0.90±0.15	0.72	0.77	0.81	0.85	0.89	0.93	0.97	1.02	1.10
**30–39**	196	0.93±0.18	0.69	0.76	0.84	0.87	0.93	0.97	1.00	1.06	1.15
**40–49**	258	0.96±0.18^D^	0.75	0.81	0.87	0.91	0.96	0.99	1.03	1.08	1.17
**50–59**	437	0.99±0.18^C^	0.77	0.86	0.91	0.95	0.99	1.03	1.07	1.12	1.19
**60–69**	440	0.99±0.16^C^	0.80	0.87	0.91	0.95	0.98	1.02	1.06	1.10	1.19
**70–79**	183	1.36±0.22^A^	1.13	1.20	1.26	1.31	1.35	1.39	1.44	1.49	1.63
	**Men**										
**Age (yr)**	n	X ± SD	90^th^	80^th^	70^th^	60^th^	50^th^	40^th^	30^th^	20^th^	10^th^
**20–29**	384	1.15±0.23	0.86	0.95	1.03	1.09	1.14	1.18	1.26	1.33	1.43
**30–39**	104	1.27±0.22^D^	1.02	1.14	1.17	1.20	1.25	1.32	1.37	1.44	1.58
**40–49**	145	1.36±0.53^C^	1.05	1.18	1.23	1.33	1.44	1.50	1.54	1.59	1.66
**50–59**	214	1.39±0.23^C^	1.11	1.18	1.28	1.33	1.38	1.44	1.49	1.55	1.65
**60–69**	236	1.43±0.27^B^	1.10	1.19	1.27	1.35	1.43	1.48	1.55	1.62	1.75
**70–79**	168	1.34±0.27^E^	1.05	1.15	1.22	1.26	1.31	1.38	1.45	1.52	1.67

^A^ Significantly different than 20–29, 30–39, 40–49, 50–59, 60–69; p<0.05.

^B^ Significantly different than 20–29, 30–39, 40–49; p<0.05.

^C^ Significantly different than 20–29, 30–39; p<0.05.

^D^ Significantly different than 20–29; p<0.05.

^E^ Significantly different than 20–29, 30–39, 60–69.

## Discussion

The ability to derive meaningful interpretations of the results from whole-body and regional DXA scans obtained with GE-Healthcare system has been challenging as representive reference values have not been available. Presently the only DXA-derived reference values for fat mass variables in adults were derived from the 1999–2004 NHANES dataset, which used measurements directly obtained from the Hologic QDR 4500A system. The current study provides the first set of reference values generated directly from measurements of %BF, FMI, trunk/limb and trunk/leg ratio obtained using the GE-Healthcare DXA systems.

The most commonly reported body composition variable is total %BF. The sex-specific data from this study cohort with DXA measures concurs with known literature showing median values for %BF in women are higher than seen in men [4, 24–26]. The current results showed a progressive increase (~ 2% per decade) in median %BF from the 20–29 year age group through the 50–59 year age group in both men and women, which was similar to the age group increase in %BF reported by Kelly et al. in Caucasian men and women [4]. The largest increase, ~5% per decade, was between the 20–29 to 30–39 year age group. The change in median value was <1% per decade for the 60–69 and 70–79 year age groups.

Comparison of the sex and age specific median values between this cohort measured with GE-Healthcare DXA and the NHANES cohort measured with Hologic DXA, in general showed similar values. However, for the youngest age group (20–29 years) the NHANES data reported medians of 24.0, 20.8, and 25.1% for whites, blacks, and Mexican-American men respectively compared to the 20.2% in the current study. This was similar in data for women with the NHANES cohort having medians of 35.5, 36.9, and 38.0% for whites, blacks, and Mexican-Americans respectively, compared to the 29.9% in the current study. Comparisons of sex-specific median values between the two cohorts for the other ages groups were generally around 1%, which is similar to the absolute difference reported by Shepherd et al of 1.4% [5]. The exception was for the 70–79 year old age group in women where the current study had a median value of 39.6% compared to the NHANES values of 43.0, 42.9, and 43.4% for whites, blacks, and Mexican-American women respectively. Thus, although there was general congruency of DXA values between the two manufacturers, there are distinct differences which suggest the need for brand-specific normative ranges for these measurements.

We also compared the reference values from this cohort to those developed by Fan et al. from a previously validated cross-calibrated equation [16] to convert measurements from the Hologic for use with the GE-Healthcare models. The median %BF at each age group were lower in women in the current study (relative differences of 1.3 to 8.2%) compared to the values from Fan et al. In men, differences in median %BF between the current study and Fan et al. ranged from -4.3 to 0.5%. Thus, it appears the use of the GE reference values derived from the cross-calibrated equation may overestimate %BF in women and underestimate %BF in men. This finding emphasizes the important of the reference values developed from direct measures with the GE-Healthcare models for accurate classification, rather than use of cross-calibrated equations.

DXA is capable of separating body mass into fat and fat-free components, thereby permitting the evaluation of fat mass without the confounding influence of other tissue constituents. FMI evaluates only the fat mass component of body mass without the interference from other body components, such as excess muscle, and is useful as a measure of abnormally low or high fat mass. Data from our study sample reveal that median FMI increases with age up until the 50–59 year age group in both men and women. The reference ranges derived from NHANES showed similar findings in women (increasing until approximately 65 years of age); but in men the median FMI continued to increase up until 80 years of age [4]. However, for the youngest age group (20–29 years) the NHANES data reported medians of 6.2, 5.2, and 6.4 kg·m⁻^2^, for whites, blacks, and Mexican-American men respectively compared to 5.0 kg·m⁻^2^ in the current study. This was similar in data for women with the NHANES cohort having medians of 8.7, 10.4, and 10.2 kg·m⁻^2^, for whites, blacks, and Mexican-Americans respectively, compared to the 6.6 kg·m⁻^2^ in the current study. Differences in age and sex-specific medians between the white population in NHANES and the current cohort ranged between -1.4 to 3.0 kg·m⁻^2^. Using the FMI classification ranges developed in the Kelly et al. study, the prevalence rates for overweight and obesity in the current study were 31.6% (FMI> 6 kg·m⁻^2^) and 31.7% (FMI>9 kg·m⁻^2^) for men. In women, the prevalence rates were 26.8% (FMI > 9 kg·m⁻^2^) for overweight and 26.4% (FMI>13 kg·m⁻^2^) for obesity. When compared to overweight and obesity prevalence rates estimated using BMI, in men the prevalence rate for overweight was higher (40.6%) (BMI ≥ 25 kg·m⁻^2^), but was lower for obesity (26.3%) (BMI ≥30 kg·m⁻^2^). In women the prevalence rates determined from BMI were slightly lower for both overweight (26.7%) and obesity (25.0%). These results indicate that BMI may overestimate adiposity in men with more lean mass and underestimate adiposity in those with excess fat mass, compared to FMI which provides a better indication of adiposity. Thus FMI as obtained from DXA measurements may provide clinicians and researchers with a useful tool to accurately identify those that are overweight/obese compared to BMI which commonly misclassifies individuals with excess lean mass.

Fat distribution is an important factor in risk classification. Abdominal fat mass, as reflected in the android and trunk fat mass measurements, is strongly associated with risk factors for cardiovascular disease and metabolic syndrome. In the current study, men had greater A/G, trunk/limb, and trunk/leg fat ratios than women at each decade. This is a result of men carrying higher amounts of android fat mass on average compared to women [27]. The trend of each ratio increasing with age in the current study was similar to findings by Kelly et al. However, the median trunk/limb and trunk/leg ratios, for both sexes across all age groups, were higher in the current study than that observed previously (trunk/limb differences men: 0.24 to 0.45, women: 0.12 to 0.18); (trunk/leg differences men: 0.21 to 0.38, women: 0.08 to 0.45) [4]. This is likely attributable to differences in the measurement technology of the two different DXA manufacturers. Shepherd et al. found differences between DXA manufacturers (Hologic and GE-Healthcare models) in sub-regional trunk %BF and leg %BF, with measurements from the GE-Healthcare models being higher in trunk %BF but lower in leg % BF compared to Hologic systems (38.5% vs. 33.4% and 32.3% vs. 33.1%, respectively). The higher trunk % BF and lower leg % BF measured by the GE-Healthcare models would cause the trunk/leg ratio to be elevated compared to Hologic measurements. Additionally, in the current study there was a disjointed increase seen for the trunk/leg ratio in men between ages 40 to 50 and in women between 60 to 70 years. Although there is no clear definitive explanation for this increase, in women it may be likely due to sex-specific hormonal changes with aging that lead to higher amounts of visceral fat accumulation [28, 29]. This should be explored in other cohorts to determine if similar changes occur in both men and women.

This study is not without limitations. It is important to note that no formal statistical comparisons were performed between the two cohorts; thus these comparisons of median values are based solely on observation. We should note that the current study’s population was similar to the NHANES cohort in distribution of age, sex distribution (approximately 53% vs. 62% women, in NHANES and current study, respectively), and mean BMI for both sexes (men 28.0 kg·m⁻^2^vs. 27.9 kg·m⁻^2^; women 27.3 kg·m⁻^2^vs. 28.2 kg·m⁻^2^, respectively), which were considered overweight for both sexes. Therefore similarly to the NHANES dataset, these data reflect the increasing prevalence of overweight and obesity of the population. The NHANES cohort had more ethnic distribution (49% white, 19% black, 23% Mexican-American, and 9% other) compared to the current study which was 95% white. As ethnic variation in body composition has been documented in the literature [30, 31], these reference ranges may not be an accurate representation for all ethnic groups. This study focused solely on fat mass variables, even though the importance of lean mass on health and well-being is well documented. Thus, manufacturer-specific reference values for lean mass variables and indices is also warranted and will be reported in a separate report. This study had several strengths including using pooled data from two laboratories and a subject group with a wide-range of characteristics including age, BMI, and physical activity levels.

## Conclusion

The value of body composition measurements as a tool to understand health and disease is growing in importance. DXA scans provide a high-quality measure of a wide-range of body composition measures, however, the interpretation of DXA data has been limited by lack of reference values. The results from this study provide reference values for %BF, FMI, fat mass ratios of A/G, trunk/limb, and trunk/leg using the GE-Healthcare models. These reference values will provide GE-Healthcare DXA system users with the ability to derive meaningful interpretation of results from whole-body and regional DXA scans. As a result, the proposed reference values will also help effectively identify those who are at an increased risk for chronic diseases associated with increased adiposity.

## Supporting information

S1 Fig Percent body fat (%) vs. age in women. Lines indicate 3^rd^ (black), 50^th^ (red), and 97^th^ (green) percentiles.
(PDF)

S2 Fig Percent body fat (%) vs. age in men. Lines indicate 3^rd^ (black), 50^th^ (red), and 97^th^ (green) percentiles.
(PDF)

S3 Fig Fat mass index (kg·m⁻^2^) vs. age in women. Lines indicate 3^rd^ (black), 50^th^ (red), and 97^th^ (green) percentiles.
(PDF)

S4 Fig Fat mass index (kg·m⁻^2^) vs. age in men. Lines indicate 3^rd^ (black), 50^th^ (red), and 97^th^ (green) percentiles.
(PDF)

S5 Fig Android-to-Gynoid ratio vs. age in women. Lines indicate 3^rd^ (black), 50^th^ (red), and 97^th^ (green) percentiles.
(PDF)

S6 Fig Android-to-Gynoid ratio vs. age in men. Lines indicate 3^rd^ (black), 50^th^ (red), and 97^th^ (green) percentiles.
(PDF)

S7 Fig Trunk-to-Limb ratio vs. age in women. Lines indicate 3^rd^ (black), 50^th^ (red), and 97^th^ (green) percentiles.
(PDF)

S8 Fig Trunk-to-Limb ratio vs. age in men. Lines indicate 3^rd^ (black), 50^th^ (red), and 97^th^ (green) percentiles.
(PDF)

S9 Fig Trunk-to-Leg ratio vs. age in women. Lines indicate 3^rd^ (black), 50^th^ (red), and 97^th^ (green) percentiles.
(PDF)

S10 Fig Trunk-to-Leg ratio vs. age in men. Lines indicate 3^rd^ (black), 50^th^ (red), and 97^th^ (green) percentiles.
(PDF)
