# Supplementary material for: Reference standards for body fat measures using GE dual energy x-ray absorptiometry in Caucasian adults
Source: PLoS One. 2017 Apr 7;12(4):e0175110. doi: 10.1371/journal.pone.0175110 (PMC5384668; doi:10.1371/journal.pone.0175110)
Supplement: S1 Fig — Lines indicate 3rd (black), 50th (red), and 97th (green) percentiles. (PDF) [file pone.0175110.s001.pdf]

Figure S1. Percent body fat vs. age in women

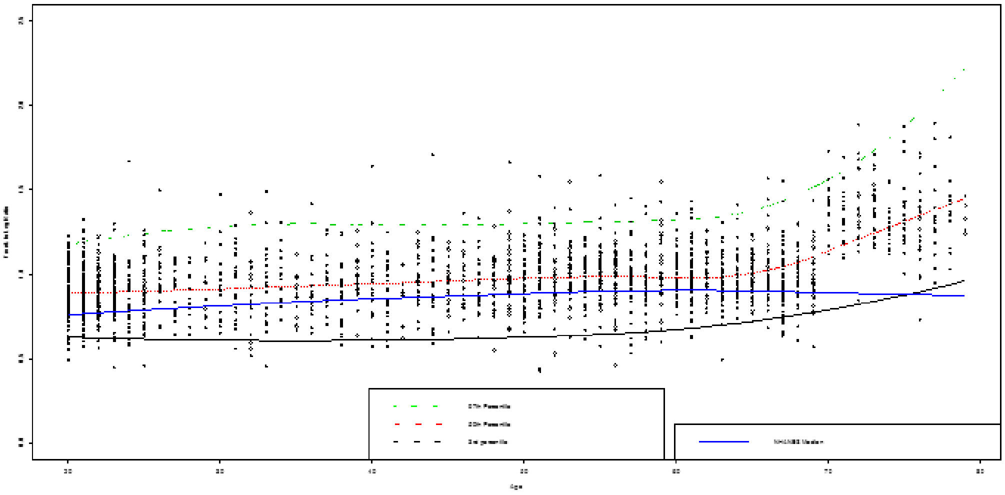

Solid black, red, and green lines indicate 3rd, 50th, and 97th percentiles
Solid blue line indicates the median values from the NHANES cohort
L (box cox transfer), M (mean), and S (coefficient of variation) values equal 0.32, 39.0, and 26.1, respectively.
