# Supplementary material for: Reference standards for body fat measures using GE dual energy x-ray absorptiometry in Caucasian adults
Source: PLoS One. 2017 Apr 7;12(4):e0175110. doi: 10.1371/journal.pone.0175110 (PMC5384668; doi:10.1371/journal.pone.0175110)
Supplement: S4 Fig — Lines indicate 3rd (black), 50th (red), and 97th (green) percentiles. (PDF) [file pone.0175110.s004.pdf]

Figure S4. Fat mass index vs. age in men

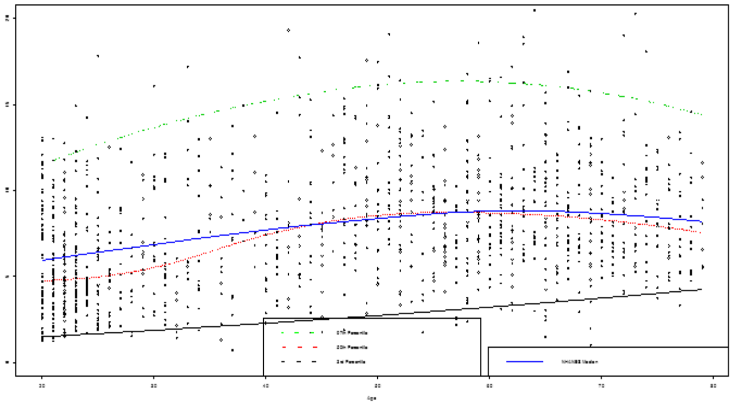

Solid black, red, and green lines indicate 3rd, 50th, and 97th percentiles
Solid blue line indicates median value from NHANES cohort
L (box cox function), M (mean), and S (coefficient of variation) values are 0.6, 7.2, and 46.8, respectively.
