# Supplementary material for: Reference standards for body fat measures using GE dual energy x-ray absorptiometry in Caucasian adults
Source: PLoS One. 2017 Apr 7;12(4):e0175110. doi: 10.1371/journal.pone.0175110 (PMC5384668; doi:10.1371/journal.pone.0175110)
Supplement: S6 Fig — Lines indicate 3rd (black), 50th (red), and 97th (green) percentiles. (PDF) [file pone.0175110.s006.pdf]

Figure S6. Android-to-Gynoid ratio vs. age in men

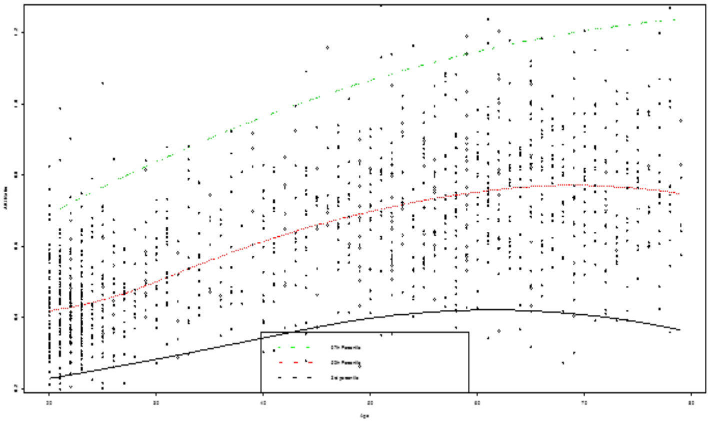

Solid black, red, and green lines indicate 3rd, 50th, and 97th percentiles
L (box cox function), M (mean), and S (coefficient of variation) values are 0.6, 0.6, and 35.5, respectively.
